# The risk of failure of subcutaneous implantable cardioverter defibrillator therapy: from PRAETORIAN score to clinical practice

**DOI:** 10.1093/europace/euaf011

**Published:** 2025-01-21

**Authors:** Matteo Ziacchi, Luca Ottaviano, Luca Checchi, Stefano Viani, Gerardo Nigro, Valter Bianchi, Silvana De Bonis, Paolo De Filippo, Pietro Francia, Antonio Rapacciuolo, Gennaro Vitulano, Giovanni Battista Perego, Vincenzo Schillaci, Carlo Lavalle, Federico Migliore, Ennio C L Pisanò, Paolo Compagnucci, Pietro Palmisano, Gianluca Botto, Roberto Rordorf, Mariolina Lovecchio, Sergio Valsecchi, Mauro Biffi

**Affiliations:** Institute of Cardiology, IRCCS Azienda Ospedaliero Universitaria di Bologna, Via Massarenti n. 9, Bologna 40138, Italy; Arrhythmia and Electrophysiology Unit, Cardiothoracic Department IRCCS Galeazzi—S. Ambrogio, Milan, Italy; Electrophysiology Unit, Careggi University Hospital, Florence, Italy; Second Cardiology Division, Cardio-Thoracic and Vascular Department, University Hospital of Pisa, Pisa, Italy; Department of Translational Medical Sciences, University of Campania ‘Luigi Vanvitelli’, Naples, Italy; ‘U.O.C. di Cardiologia-UTIC’ Department—Azienda Ospedaliera ‘dei Colli’—Ospedale Monaldi, Naples, Italy; Division of Cardiology, Castrovillari Hospital, Cosenza, Italy; Cardiac Electrophysiology and Pacing Unit, Papa Giovanni XXIII Hospital, Bergamo, Italy; Cardiology, Department of Clinical and Molecular Medicine, Sant’Andrea Hospital, University Sapienza, Rome, Italy; Department of Advanced Biomedical Sciences, Federico II University of Naples, Naples, Italy; Cardiology Unit, Ospedali Riuniti San Giovanni di Dio e Ruggi D’Aragona, Salerno, Italy; Division of Cardiology, Istituto Auxologico Italiano, Milan, Italy; Cardiology Unit, Casa Di Cura Montevergine, Mercogliano, Italy; Dipartimento di Scienze Cliniche Internistiche, Anestesiologiche e Cardiovascolari Università Sapenza Roma, Rome, Italy; Department of Cardiac, Thoracic Vascular Sciences and Public Health, University of Padova, Padua, Italy; Cardiology and Intensive Care Unit, Ospedale ‘V. Fazzi’, Lecce, Italy; Cardiology and Arrhythmology Clinic, Università Politecnica delle Marche, Ancona, Italy; Cardiology Unit, ‘Card. G. Panico’ Hospital, Tricase, Italy; U.O. Electrophysiology, ASST Rhodense, Rho-Garbagnate Milanese (MI), Italy; Arrhythmias and Electrophysiology Unit, Division of Cardiology, IRCCS Policlinico S. Matteo, Pavia, Italy; Cardiac Rhythm Management, Boston Scientific, Milan, Italy; Cardiac Rhythm Management, Boston Scientific, Milan, Italy; Institute of Cardiology, IRCCS Azienda Ospedaliero Universitaria di Bologna, Via Massarenti n. 9, Bologna 40138, Italy

**Keywords:** Implantable defibrillator, Subcutaneous, Defibrillation test, Conversion

## Abstract

**Aims:**

The subcutaneous implantable cardioverter defibrillator (S-ICD) is an alternative to traditional ICDs. The PRAETORIAN score, based on chest radiographs, has been validated to predict the probability of successful S-ICD defibrillation testing by assessing factors like fat thickness between the coil and sternum and generator placement. This study evaluated the correlation between the PRAETORIAN score and clinical characteristics, as well as implantation variables.

**Methods and results:**

We retrospectively analysed data from 1253 patients who had undergone implantation of an S-ICD across 33 centres. The intermuscular positioning of the pulse generator was adopted in all patients. Post-implantation posterior–anterior and lateral chest radiographs were analysed to calculate the PRAETORIAN score. A total of 95.7% of patients had a PRAETORIAN score < 90, indicative of a low risk of conversion failure. Body mass index (BMI) was the only independent predictor of a score ≥ 90, and all patients with BMI < 25 kg/m^2^ (normal weight or underweight) had a score < 90. The intermuscular positioning technique resulted in optimal posterior placement of the device in all patients and significant sub-generator fat in only 3% of cases. A shock impedance value > 88 Ohm enabled to detect a PRAETORIAN score ≥ 90 with 98% (95% CI 97–99%) negative predictive value.

**Conclusion:**

In contemporary practice, the PRAETORIAN score can be simplified. By adopting an intermuscular approach, two of the three steps of the score—evaluating the adequate posterior positioning of the generator and measuring the sub-generator fat—become superfluous, and impedance may serve as a reliable surrogate of sub-coil fat thickness. Furthermore, our data suggest that for non-obese patients, a favourable PRAETORIAN score is assured, making the score evaluation potentially unnecessary.

**Clinical trial registration:**

URL: http://clinicaltrials.gov/ Identifier: NCT02275637.

## Graphical Abstract

**graphical abstract:**
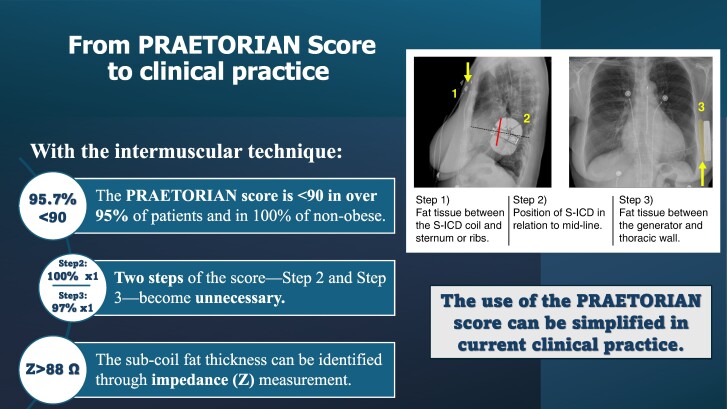

**What’s new?**

- With the optimized intermuscular technique for generator placement, the PRAETORIAN score is <90 (low risk of conversion failure) in over 95% of patients and consistently favourable in non-obese patients.
- By adopting an intermuscular approach, two of the three steps of the score—evaluating the adequate posterior positioning of the generator via posterior–anterior chest radiograph and measuring the sub-generator fat—become potentially unnecessary.
- The sub-coil fat thickness can be accurately identified through impedance measurement provided by the device, which serves as a reliable surrogate.

## Introduction

The subcutaneous implantable cardioverter defibrillator (S-ICD) is a proven alternative to the traditional implantable cardioverter defibrillator (ICD) and was shown to reduce transvenous lead-related complications.^1–3^ In contrast to the transvenous ICD, the S-ICD is a completely extra thoracic device, implanted with the generator on the left side of the thoracic wall and the lead on the sternum. Correct implant positioning is crucial to guarantee successful shock efficacy.^4^ Optimal implantation of the S-ICD requires minimizing the amount of adipose tissue between the coil and the sternum and between the generator and the thorax. Moreover, the antero-posterior position of the S-ICD generator needs to be on or posterior to the midline. The PRAETORIAN score is a chest radiograph-based method that assesses these determinants of the defibrillation threshold and has been retrospectively validated to predict the probability of successful S-ICD defibrillation testing (DT).^5^ Functional DT is currently recommended on S-ICD implantation.^6^ However, the Randomised Trial of S-ICD Implantation With and Without Defibrillation Testing (PRAETORIAN-DFT) is currently ongoing to investigate whether S-ICD implantation without DT but with PRAETORIAN score calculation is non-inferior to implantation with testing, with regard to first shock efficacy in spontaneous events.^7^ Pending on the long-term outcomes of this trial, we wondered about the possible use of the score in clinical practice in relation to the clinical characteristics of the patient and the implantation variables. Indeed, the score is strictly dependent on the physical characteristics of the S-ICD candidate, and the adoption of new implantation techniques^8^ might affect optimal chest radiograph position of the system.

## Methods

### Study design

From 2018 to 2023, consecutive patients undergoing de-novo implantation of an S-ICD (Boston Scientific Inc., Natick, MA, USA) were enrolled at 33 Italian centres (see Appendix). Data were collected at the study centres within the framework of a prospective registry (clinical trial registration: URL: http://clinicaltrials.gov/Identifier: NCT02275637). The Institutional Review Boards approved the study, and all patients provided written informed consent for data storage and analysis. Baseline assessment comprised the collection of demographic data and medical history, clinical examination, 12-lead electrocardiogram, and echocardiographic evaluation. In the present analysis, we included only patients with available post-implantation posterior–anterior and lateral chest radiographs.

### Implantation procedure

**Figure 1:**
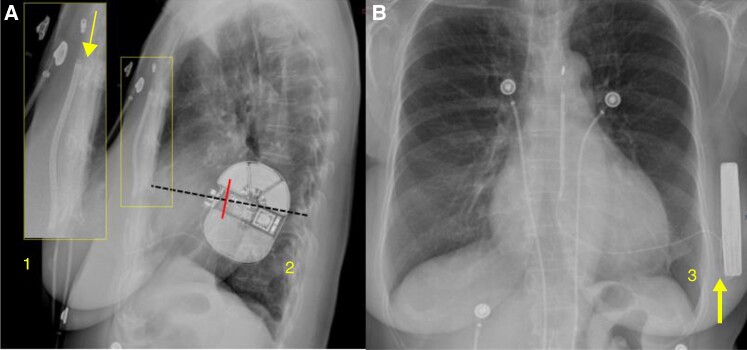
Chest radiograph analysis according to the PRAETORIAN score. In *A*, the thickness of the adipose tissue is ≤1 coil width. This corresponds to 30 points in the score system (Step 1 of the PRAETORIAN score) and low predicted risk of shock failure. The lateral view also allows to evaluate the generator placement in relation to the midline (Step 2 of the PRAETORIAN score). The S-ICD generator is in an optimal position, posterior to the midline; the score is, therefore, multiplied by 1. In *B*, the postero-anterior chest radiograph view allows to determine the amount of sub-generator fat (Step 3 of the PRATEORIAN score). As a reference, the generator width is used. In the example, <1 generator width of fat tissue is observed between the nearest point of the generator and the thoracic wall. The score is multiplied by 1.

Before implantation, the adequacy of S-ICD sensing was verified through surface screening based on a dedicated ECG morphology tool.^9^ The pulse generator was positioned between the anterior surface of the serratus anterior and the posterior surface of the latissimus dorsi muscles (intermuscular positioning). The two-incision technique was adopted for lead deployment, i.e. the defibrillation lead was tunnelled from the parasternal incision, just above the level of the xiphoid process, to the lateral pocket, and then vertically positioned along the sternum by means of a peel-away sheath introducer.^10^ At the end of the procedure, the decision to perform DT was left to the discretion of the implanting physician. Defibrillation testing was considered successful if the device detected and terminated the induced ventricular fibrillation by using ≤65 J shock energy. Post-implantation posterior–anterior and lateral chest radiographs were analysed in order to calculate the PRAETORIAN score, as previously described^5^ (*Figure 1*). In brief, the thickness of the adipose tissue between the coil and the sternum is assessed (from 30 to 150 points), the posterior or anterior positioning of the generator is determined (multiplication factor: from ×1 to ×4), and the sub-generator fat is measured (multiplication factor: from ×1 to ×1.5). In the case of a score of ≥90, 40 points are subtracted for patients with a body mass index (BMI) ≤ 25 kg/m^2^. The final score indicates the risk of conversion failure according to the following cut-off values: <90 points (low risk), between ≥90 and <150 points (intermediate risk), and ≥150 (high risk).

### Statistical analysis

Descriptive statistics are reported as means ± SD. Categorical variables are reported as percentages. One-way ANOVA was used to test for differences of continuous variables. Differences in proportions were compared by means of χ^2^ analysis. Logistic regression analysis was used to determine the association between PRAETORIAN scores and clinical characteristics and implantation variables, and to estimate the odds ratios (ORs) and the 95% confidence intervals (CIs). All variables displaying a *P* < 0.1 were included in a multivariable binary logistic regression analysis. A receiver operating characteristic (ROC) curve analysis was conducted to assess the performance of the shock impedance in predicting the thickness of the adipose tissue between the coil and the sternum; the value resulting in the maximum sum of sensitivity and specificity on the curve was regarded as the optimal cut-off. A *P* value of <0.05 was considered significant for all tests. All statistical analyses were performed by means of R: a language and environment for statistical computing (R Foundation for Statistical Computing, Vienna, Austria).

## Results

### Study population

During the observation period, 1710 patients received an S-ICD at the 33 study centres and 1253 (73%) had posterior–anterior and lateral chest radiographs of adequate quality for PRAETORIAN score assessment. *Table 1* shows the baseline clinical variables of the study population.

**Table 1:** Demographics and baseline clinical characteristics

Parameter	*n* = 1253
Male gender, *n* (%)	955 (76)
Age, years	50 ± 15
Body mass index, kg/m^2^	26 ± 4
LV ejection fraction, %	44 ± 16
Ischaemic/non-ischaemic dilated cardiomyopathy, *n* (%)	681 (54)
Hypertrophic cardiomyopathy, *n* (%)	186 (15)
Arrhythmic syndromes, *n* (%)	386 (31)
Chronic kidney disease, *n* (%)	100 (8)
Diabetes, *n* (%)	163 (13)

LV, left ventricular.

### PRAETORIAN score and clinical variables

The PRAETORIAN score was <90 (low risk of conversion failure) in 1199 (95.7%) patients. On univariate analysis (*Table 2*), the baseline clinical variables that showed a significant association with a PRAETORIAN score ≥ 90, i.e. intermediate and high risks of conversion failure, were BMI as a continuous variable and diabetes. On multivariate analysis, only higher BMI confirmed as independent predictor. Specifically, all 583 patients with BMI < 25 kg/m^2^ (normal weight or underweight) had a PRAETORIAN score < 90.

**Table 2:** Regression analysis of factors associated with PRAETORIAN score ≥ 90 (intermediate and high risks)

	Univariate analysis			Multivariate analysis		
*n* = 1253	OR	95% CI	*P*	OR	95% CI	*P*
Male gender	1.01	0.52–2.00	0.967	–	–	–
Age	1.01	0.99–1.03	0.153	–	–	–
Body mass index	1.24	1.17–1.30	<0.001	1.24	1.17–1.31	<0.001
Left ventricular ejection fraction	0.99	0.98–1.01	0.522	–	–	–
Ischaemic/non-ischaemic dilated cardiomyopathy	1.14	0.66–1.98	0.645	–	–	–
Hypertrophic cardiomyopathy	1.32	0.65–2.67	0.439	–	–	–
Arrhythmic syndromes	0.70	0.37–1.33	0.276	–	–	–
Chronic kidney disease	1.18	0.46–3.02	0.737	–	–	–
Diabetes	2.62	1.35–5.07	0.004	1.49	0.72–3.08	0.277

### PRAETORIAN score and generator placement

**Figure 2:**
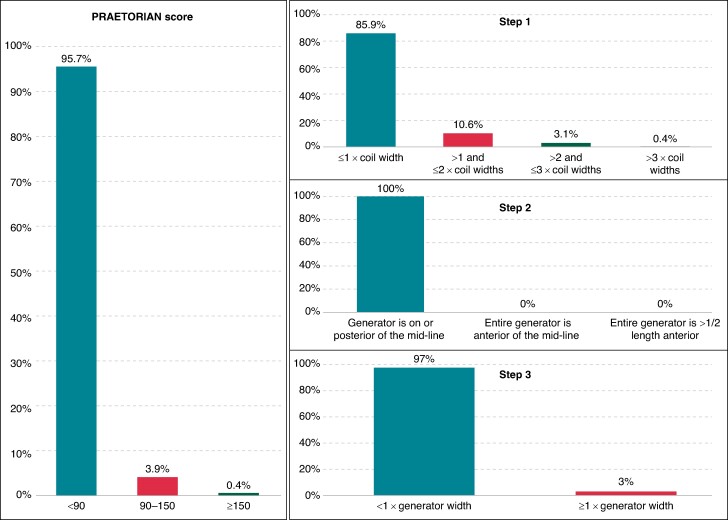
Distribution of the overall PRAETORIAN score and the three steps: (Step 1) thickness of the adipose tissue between the coil and the sternum, measured by using the coil width as a reference; (Step 2) position of the generator in relation to the midline; (Step 3) amount of fat tissue between the nearest point of the generator and the thoracic wall, measured by using the generator width as a reference.

In all patients, the intermuscular positioning of the generator resulted in optimal posterior placement of the device in relation to the midline (Step 2, *Figure 2*). Moreover, the amount of fat tissue between the generator and the thoracic wall was less than the generator width in 1215 (97%) patients (Step 3). When calculating the PRAETORIAN score, the combination of these two conditions determined a total multiplication factor ≤ 1.5 from Steps 2 and 3 (×1.5 only in 3% of cases).

### PRAETORIAN score and coil placement

Adopting the two-incision technique for lead deployment, the thickness of the adipose tissue between the coil and the sternum was ≤1 coil width in 1076 (85.9%) patients. According to the Step 1 of the PRAETORIAN score calculation scheme, this resulted in 30 points. That is, a final PRAETORIAN score < 90 even in the case of substantial sub-generator fat (a ×1.5 multiplication factor from Step 3).

### Shock impedance

**Figure 3:**
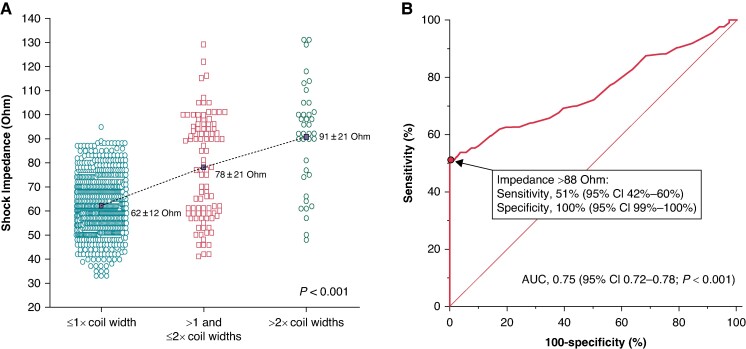
(*A*) Stepwise increase of shock impedance according to the thickness of the adipose tissue between the coil and the sternum. (*B*) Receiver operating characteristic curve analysis of the shock impedance for the prediction of thickness of the adipose tissue between the coil and the sternum > 1 coil width (PRAETORIAN score Step 1: ≥60 points). Sensitivity and specificity were 51% (95% confidence interval 42–60%) and 100% (95% confidence interval 99–100%), respectively, at shock impedance > 88 Ohm.

The analysis of factors associated with sub-coil fat was performed in the subgroup of 837 patients with an available shock impedance value, obtained from either the DT or a shock impedance test. The regression analysis identified BMI and shock impedance as factors associated with >1 coil width of sub-coil fat (*Table 3*). However, only shock impedance was confirmed as independent predictor, and showed a significant stepwise increase according to sub-coil fat (*Figure 3*). On the basis of the ROC curve analysis of shock impedance values for the prediction of >1 coil width of sub-coil fat, the area under curve was 0.75 (95% CI 0.72–0.78; *P* < 0.001). The cut-off that maximized sensibility and specificity was 88 Ohm. It enabled >1 coil width of sub-coil fat to be detected with 51% (95% CI 42–60%) sensitivity and 100% (95% CI 99–100%) specificity (*Figure 3*).

**Table 3:** Regression analysis of factors associated with >1 coil width of fat tissue between the S-ICD coil and the sternum ribs (Step 1 ≥ 60)

	Univariate analysis			Multivariate analysis		
*n* = 837	OR	95% CI	*P*	OR	95% CI	*P*
Male gender	0.94	0.64–1.40	0.755	–	–	–
Age	1.00	0.99–1.01	0.882	–	–	–
Body mass index	1.11	1.08–1.15	<0.001	1.03	0.98–1.08	0.265
Left ventricular ejection fraction	1.00	0.99–1.01	0.442	–	–	–
Shock impedance	1.08	1.07–1.10	<0.001	1.08	1.07–1.10	<0.001

Among the 837 patients with shock impedance values, the PRAETORIAN score was ≥90 in 47 (5.6%) patients. A shock impedance value > 88 Ohm (measured in 74 cases) enabled to detect a PRAETORIAN score ≥ 90 with 98% (95% CI 97–99%) negative predictive value and 46% (95% CI 35–59%) positive predictive value (see Supplementary material online, *Table S1*). The results were similar in the subgroup of 434 patients with BMI ≥ 25 kg/m^2^ (all the remaining ones had a PRAETORIAN score < 90). Indeed, in this subgroup, a shock impedance value > 88 Ohm (measured in 59 cases) detected a PRAETORIAN score ≥ 90 with 96% (95% CI 93–98%) negative predictive value and 55% (95% CI 41–68%) positive predictive value.

### Defibrillation testing

After implantation, the DT was performed in 825 patients. Cardioversion was successful at a ≤65 J shock energy in 793 (96.1%) patients. In total, 778 patients had a PRAETORIAN score < 90 of whom 27 (3.5%) had a failed DT. Forty-seven patients had a score ≥ 90 of whom 5 (10.6%) had a failed testing. This resulted in 97% (95% CI 95–98%) negative predictive value and 11% (95% CI 4–23%) positive predictive value (see Supplementary material online, *Table S2*). In the 32 patients with failed DT despite intermuscular device implantation, the generator was never anterior to the midline (0%) and the sub-generator fat was rarely substantial (9%, 3/32). The width of sub-coil fat was >1 coil in 22% of patients (7/32), and 69% (22/32) of them were overweight/obese. Defibrillation testing failure according to BMI and shock impedance values is reported in Supplementary material online, *Table S3*.

## Discussion

The PRAETORIAN score was developed to assess the primary determinants of the S-ICD defibrillation threshold and integrate them into a single index. It has been validated for predicting the probability of a successful DT,^5^ and research is ongoing to evaluate its potential as an alternative to the DT during implantation.^7^ The technique for S-ICD implantation has evolved significantly over time. Unlike the methods used during the development and validation of the PRAETORIAN score, the current practice widely employs an intermuscular pocket for the generator and avoids the superior parasternal incision by using a specialized introducer.^8,10^ These advancements in implantation techniques and increased operator experience, which have led to improved implant outcomes, may have affected the value of the PRAETORIAN score.

The PRAETORIAN score was initially validated using data from the IDE study cohort, which included the first S-ICD implants performed by many operators.^11^ At that time, 87% of implants had a PRAETORIAN score < 90. In a recent sub-analysis of the PRAETORIAN-DFT trial,^12^ which involved 97% of procedures using a two-incision technique and 76% of generators implanted in an intermuscular pocket, 92% of implants had a PRAETORIAN score < 90. In our study, which included a large sample of S-ICD procedures all conducted with an intermuscular two-incision approach, we observed a very high (95.7%) proportion of implants with a PRAETORIAN score indicative of a low risk of conversion failure. Notably, this safety criterion was consistently met in normal-weight patients undergoing implantation.

Previous studies suggested that the intermuscular technique might prevent anterior malpositioning of the S-ICD and reduce fat interposition between the generator and the chest.^13^ Our findings support this, showing optimal posterior placement of the device relative to the midline in all patients and significant sub-generator fat in only 3% of cases. Consequently, radiographic verification of the generator’s position may be unnecessary, simplifying the scoring procedure to assessing sub-coil fat thickness alone.

In our study cohort, the thickness of the adipose tissue between the coil and the sternum was ≤1 coil width in 85.9% of patients. The two-incision technique, which employs a dedicated sheath introducer for deep coil implantation, contributes to this result. Our analysis identified shock impedance as an independent predictor of substantial sub-coil fat and demonstrated a significant stepwise increase in impedance with increased sub-coil fat. This supports previous studies,^4,14^ which indicated that sub-coil fat has a greater impact on shock impedance than sub-generator fat. We found that a shock impedance value > 88 Ohm predicted a PRAETORIAN score ≥ 90 with a 98% negative predictive value, suggesting that impedance can serve as a surrogate for the PRAETORIAN score in detecting successful cardioversion.

Historically, using impedance as a predictor for defibrillation failure has been discouraged due to the potential for failed conversion despite low impedance, caused by anterior S-ICD positioning that can lead to energy shunting across the thoracic wall.^12^ Additionally, patient characteristics could contribute to higher impedance and increased defibrillation thresholds. However, with the intermuscular technique, generator malpositioning is excluded, and our results did not show any association between clinical variables and indicators of defibrillation failure. A challenge in adopting impedance as a surrogate for conversion success is the requirement to deliver a dedicated 10 J shock, which necessitates sedation or general anaesthesia, similar to the DT. Recently, the S-ICD has been upgraded to measure low voltage impedance, which correlates strongly with high-voltage measurements, thus eliminating the risks and logistical issues associated with the procedure.^15^

The possibility of omitting the DT after S-ICD implantation is motivated by the opportunity to avoid potential, albeit rare, complications,^16^ as well as to reduce logistical challenges and procedure time. Landmark trials^17–19^ have demonstrated the non-inferiority of DT omission regarding death and shock efficacy in transvenous ICDs. Consequently, current guidelines^20^ allow DT to be omitted in patients undergoing transvenous ICD implantation. Findings from clinical practice in the USA and Europe^20–24^ have shown high rates of successful conversion on DT and a very high defibrillation safety margin with current S-ICD devices delivering a maximum shock energy of 80 J.^25^ However, the results of the trials on DT omission with traditional ICDs cannot be extended to the S-ICD. Therefore, DT is still recommended for S-ICD implantation,^6^ except for patients with contraindications, such as a known intra-cardiac thrombus, atrial fibrillation or flutter without adequate systemic anticoagulation, haemodynamic instability, very poor ejection fraction, or other morbidities associated with poor outcomes. In these cases, as well as in cases of induction failure during DT (3% in the PRAETORIAN-DFT trial), the PRAETORIAN score can already be used to determine the correct positioning of the S-ICD. For other S-ICD candidates, only the PRAETORIAN-DFT trial^7^ will provide evidence on the safety of the strategy consisting of DT omission with score assessment, although adherence to the DT recommendation is already declining in clinical practice.^21,26^

The possibility of omitting the evaluation of the PRAETORIAN score in selected patients or using reliable surrogates could be valuable, especially in cases where score evaluation is not feasible. According to the recently published results of the sub-analysis of the PRAETORIAN-DFT trial,^12^ the percentage of radiographs not eligible for PRAETORIAN score calculation is high (up to 27%). Another limitation of routine PRAETORIAN score use is its post-procedural implementation, which requires early reintervention when a high risk of conversion failure is detected. This results in patient discomfort, the need for a second operating theatre access, and an increased infective risk related to pocket reopening. To overcome these limitations, a proof-of-concept study tested a method to assess the PRAETORIAN score using fluoroscopy during S-ICD placement,^27^ showing a good agreement with the score evaluated on subsequent chest X-rays.

Finally, the current study also confirms the performance of the PRAETORIAN score as a predictor for successful DT, as reported in the recently published PRAETORIAN-DFT trial analysis.^12^ The high negative predictive value (97%) supports the conclusion that a PRAETORIAN score < 90 serves as a reliable predictor for successful DT. The positive predictive value of 11% aligns with the 8.7% value found in the trial after exclusion of patients with a repositioning immediately after a failed DT. Its absolute value is low due to the overall low rate of failed DT; however, it confirms the substantial chance of unsuccessful defibrillation with a PRAETORIAN score > 90. In patients with failed DT despite a low score, only a high BMI was consistently observed. However, we lack sufficient information to identify a specific cause of failure. Potential reasons may include the patient’s clinical profile, the drug therapy, or, more generally, the stochastic nature of defibrillation.

### Limitations

This study has several limitations. Despite the prospective collection of data and consecutive enrolment of patients, the analysis was retrospective, and implantation and DT protocols varied among implanting centres. Additionally, the PRAETORIAN score was calculated by local operators, unlike the PRAETORIAN-DFT study, which used a core lab to evaluate chest radiographs. However, central evaluation does not reflect the standard approach of centres adopting the score in clinical practice. Furthermore, patients with radiographs of insufficient quality were excluded from the analysis, which may have introduced bias. Additionally, we cannot rule out that organizational factors may have prevented score calculation for some patients, limiting our ability to report the actual feasibility of score calculation. Moreover, high-voltage impedance data were not available for all patients, so the association between impedance and score values was tested on a subgroup. Similarly, the association between the score and cardioversion success was evaluated only among those who underwent DT. Lastly, this study lacks data on long-term outcomes.

### Conclusions

In summary, in this analysis of a population more than twice the size of the recently published PRAETORIAN-DFT analysis, we have shown that the use of the PRAETORIAN score can be simplified in current clinical practice. By adopting an intermuscular approach, two of the three steps of the score—evaluating the adequate posterior positioning of the generator via posterior–anterior chest radiograph and measuring the sub-generator fat—become superfluous. Operators should, therefore, focus only on assessing the sub-coil fat thickness, for which impedance serves as a reliable surrogate with an extremely high negative predictive value. Furthermore, our data suggest that for non-obese patients, a favourable PRAETORIAN score is assured, making the score evaluation potentially unnecessary at the time of implantation.

## Supplementary Material

euaf011_Supplementary_Data

## Data Availability

The experimental data used to support the findings of this study are available from the corresponding author upon reasonable request.

## Appendix

**Appendix:** List of participating centres

• ASST Rhodense, Rho-Garbagnate Milanese, Milan: G.L. Botto, F.L. Canevese, M.C. Casale;
• ASST Sette Laghi, Ospedale di Circolo e Fondazione Macchi, Varese: F. Caravati;
• Azienda Ospedaliera Universitaria Senese, Siena: A. Santoro, C. Baiocchi, R. Gentilini, S. Lunghetti;
• Clinica Montevergine, Mercogliano, Avellino: F. Solimene, G. Shopova, V. Schillaci, A. Arestia, A. Agresta;
• Fatebenefratelli Hospital, Rome: S. Bianchi, P. Rossi, F.M. Cauti;
• ‘Giovan Battista Grassi’ Hospital, Ostia, Rome; F. Ammirati, L. Santini, K. Mahfouz, C. Colaiaco;
• IRCCS Azienda Ospedaliero Universitaria di Bologna: M. Biffi, I. Diemberger, M. Ziacchi, C. Martignani, A. Angeletti, G. Massaro. A. Spadotto;
• IRCCS Fondazione Policlinico ‘S. Matteo’, Pavia: R. Rordorf, A. Vicentini, S. Savastano, B. Petracci, A. Sanzo, E. Baldi, M. Casula;
• Istituto Auxologico Italiano—IRCCS, Milan: G.B. Perego, V. Rella;
• Istituto Clinico IRCCS Galeazzi—Sant’Ambrogio, Milan: L. Ottaviano;
• Monaldi Hospital, Naples: A. D’Onofrio, V. Bianchi; V. Tavoletta, S. De Vivo;
• Ospedale ‘G. Panico’, Tricase, Lecce: P. Palmisano; M. Accogli;
• Ospedale ‘Vito Fazzi’, Lecce: E. Pisanò, G. Milanese;
• Ospedale Di Venere, Carbonara di Bari, Bari: Massimo Vincenzo Bonfantino;
• Ospedale Papa Giovanni XXIII, Bergamo: P. De Filippo; P. Ferrari; C. Leidi;
• Ospedale S. Anna e S. Sebastiano, Caserta: M. Viscusi, M. Brignoli, A. Mattera;
• Ospedale S. Donato, Arezzo: P. Notarstefano, M. Nesti, A. Fraticelli;
• Ospedale S. Andrea, Rome: P. Francia, F. Palano, C. Adduci;
• Ospedali Riuniti San Giovanni di Dio e Ruggi d’Aragona, Salerno: M. Manzo; C. Esposito, A. Giano. F. Franculli;
• Ospedali Riuniti, Reggio Calabria: A. Pangallo;
• P.O. Ferrari, Castrovillari, Cosenza: G. Bisignani, S. De Bonis;
• P.O. Santa Maria della Pietà, Nola, Naples: C. Muto
• Paediatric Cardiology Unit, Second University of Naples, Napoli: B. Sarubbi, D. Colonna, A. Correra, E. Romeo;
• Policlinico Federico II, Naples: A. Rapacciuolo, V. Liguori, A. Viggiano, T. Strisciullo;
• Policlinico Umberto I, Rome: A. Piro; C. Lavalle, M. Magnocavallo, M.V. Mariani;
• ‘San Giovanni Battista’ Hospital, Foligno: G. Savarese, C. Andreoli, L. Pimpinicchio, D. Pellegrini;
• ‘San Luca’ Hospital, Lucca: D. Giorgi, Bovenzi, F. Busoni;
• Università Politecnica delle Marche, Ancona: A. Dello Russo, M. Casella, F. Guerra, L. Cipolletta, S. Molini;
• University Hospital of Pisa, Pisa: R. De Lucia, A. Di Cori, G. Grifoni, L. Paperini, L. Segreti, E. Soldati, S. Viani, G. Zucchelli;
• University of Campania ‘Luigi Vanvitelli’, Naples: G. Nigro, V. Russo, A. Rago, E. Ammendola, A. Papa;
• University of Florence, Florence: P. Pieragnoli, G. Ricciardi, L. Checchi, L. Perrotta;
• University of Padua: F. Migliore.
