# Supplementary material for: The risk of failure of subcutaneous implantable cardioverter defibrillator therapy: from PRAETORIAN score to clinical practice
Source: Europace. 2025 Jan 21;27(2):euaf011. doi: 10.1093/europace/euaf011 (PMC11822678; doi:10.1093/europace/euaf011)
Supplement: euaf011_Supplementary_Data [file euaf011_supplementary_data.docx]

**Supplemental Table 1.** Diagnostic performance of Shock impedance >88 Ohm to detect a PRAETORIAN score ≥90.

|  | **Patients**  **(837)** | **Patients with BMI ≥25 kg/m2**  **(434)** |
| --- | --- | --- |
| Sensitivity | 68% (95% CI 53%–81%) | 68% (95% CI 53%–81%) |
| Specificity | 95% (95% CI 93%–96%) | 93% (95% CI 90%–95%) |
| Positive Predictive Value | 46% (95% CI 35%–59%) | 55% (95% CI 41%–68%) |
| Negative Predictive Value | 98% (95% CI 97%–99%) | 96% (95% CI 93%–98%) |
| *Sensitivity:* Probability of Shock impedance >88 Ohm in patients with PRAETORIAN score ≥90; *Specificity:* Probability of Shock impedance ≤88 Ohm in patients with PRAETORIAN score <90; *Positive predictive value:* Probability of PRAETORIAN score ≥90 in patients with Shock impedance >88 Ohm; *Negative predictive value:* Probability of PRAETORIAN score <90 in patients with Shock impedance ≤88 Ohm. | | |

**Supplemental Table 2.** Positive and negative predictive value of the PRAETORIAN score for the prediction of defibrillation testing failure.

| **PRAETORIAN Score** | **Patients**  **(825)** | **Successful DT**  **(793)** | **Failed DT**  **(32)** | **Score components in the 32 patients with failed DT** | | | |
| --- | --- | --- | --- | --- | --- | --- | --- |
|  |  |  |  | **Step 1** | **Step 2** | **Step 3** | **High BMI** |
| 30 | 683 | 659 | 24 | 0 | 0 | 0 | 16 |
| 45 | 7 | 6 | 1 | 0 | 0 | 1 | 0 |
| 50 | 6 | 6 | 0 | 0 | 0 | 0 | 0 |
| 60 | 82 | 80 | 2 | 2 | 0 | 0 | 1 |
| 90 | 38 | 34 | 4 | 4 | 0 | 1 | 4 |
| 135 | 6 | 5 | 1 | 1 | 0 | 1 | 1 |
| 150 | 2 | 2 | 0 | 0 | 0 | 0 | 0 |
| 225 | 1 | 1 | 0 | 0 | 0 | 0 | 0 |
| <90 | 778 | 751 | 27 (3.5%) | NPV= 97% (95%CI = 95%-98%) | | | |
| ≥90 | 47 | 42 | 5 (10.6%) | PPV= 11% (95% CI 4%-23%) | | | |
| *Step 1*: >1 coil width of sub-coil fat; *Step 2*: Generator anterior to the midline; *Step 3*: >1 generator width of sub-generator fat; *High BMI*: BMI ≥25 kg/m2; CI: Confidence interval; DT: Defibrillation test; NPV: Negative predictive value; PPV: Positive predictive value. | | | | | | | |

**Supplemental Table 3.** Defibrillation testing failure according to body mass index and shock impedance values.

|  | **Patients**  **(825)** | **Successful DT**  **(793)** | **Failed DT**  **(32)** | **p-value** |
| --- | --- | --- | --- | --- |
| BMI <25 kg/m2 | 390 | 380 | 10 (2.6%) |  |
| BMI ≥25 kg/m2 | 435 | 413 | 22 (5,1%) | 0.064 |
| Z ≤88 Ohm  Z >88 Ohm | 751  74 | 725  68 | 26 (3.5%)  6 (8.8%) | 0.048 |
| BMI: Body mass index; Z: Shock impedance. | | | | |
